# Speech-in-speech perception and executive function involvement

**DOI:** 10.1371/journal.pone.0180084

**Published:** 2017-07-14

**Authors:** Marcela Perrone-Bertolotti, Maxime Tassin, Fanny Meunier

**Affiliations:** 1 Univ. Grenoble Alpes, CNRS, LPNC, UMR 5105, Grenoble, France; 2 Univ. Claude Bernard Lyon I, CNRS, L2C2, Lyon, France; 3 Univ. Côte d’Azur, CNRS, BCL, Nice, France; Harvard Medical School, UNITED STATES

## Abstract

This present study investigated the link between speech-in-speech perception capacities and four executive function components: response suppression, inhibitory control, switching and working memory. We constructed a cross-modal semantic priming paradigm using a written target word and a spoken prime word, implemented in one of two concurrent auditory sentences (cocktail party situation). The prime and target were semantically related or unrelated. Participants had to perform a lexical decision task on visual target words and simultaneously listen to only one of two pronounced sentences. The attention of the participant was manipulated: The prime was in the pronounced sentence listened to by the participant or in the ignored one. In addition, we evaluate the executive function abilities of participants (switching cost, inhibitory-control cost and response-suppression cost) and their working memory span. Correlation analyses were performed between the executive and priming measurements. Our results showed a significant interaction effect between attention and semantic priming. We observed a significant priming effect in the attended but not in the ignored condition. Only priming effects obtained in the ignored condition were significantly correlated with some of the executive measurements. However, no correlation between priming effects and working memory capacity was found. Overall, these results confirm, first, the role of attention for semantic priming effect and, second, the implication of executive functions in speech-in-noise understanding capacities.

## Introduction

Speech perception rarely occurs in an optimal acoustic environment. Ecological speech perception arises in noisy contexts such as traffic or simultaneous speakers' voices in the background, typically referred to as speech-in-speech—SiS—situations or “Cocktail Party” [1]. These SiS situations degrade the information conveyed by speech, hampering the understanding of linguistic messages. The aim of the present study was to investigate the ability to access words’ semantic information in SiS situations.

Semantic processing can be evaluated using a priming paradigm in which prime and target words are semantically related and targets are recognized faster and more accurately, in comparison to a condition in which the prime and the target are semantically unrelated [2–7]. This effect is observed using the classical semantic priming paradigm (i.e., with isolated visual prime and visual target word) but also with cross-modal priming paradigm (i.e., with auditory prime words, isolated or implemented in a sentential context, and visual target word [8–12]). Moreover, a recent study [13] using a cross-modal semantic priming paradigm in a SiS situation made of 1 to 4 multi-talker babbles showed that semantic processing is dependent on prime intelligibility. The semantic priming effect was observed in the 1 and 2 talker babbles, however with 3 talker babble the priming effect was observed only when the number of semantically related voices in the babble was greater than the number of semantically unrelated voices. The authors interpreted these results in line with the Effortfulness Hypothesis [14] and suggested that with the increased difficulty in target signal extraction induced by the increased number of voices in the SiS situation, more cognitive resources are needed for formal processes such as the ones involved in processing acoustic and phonological information. This, in turn leads to a decrease in available resources for a deeper semantic processing of background words, as reveled by the absence of semantic priming effect. In line with this result, several studies have shown that SiS perception is associated with a greater cognitive load [15,16].

As SiS situations increase cognitive load, listeners’ cognitive abilities to manage this load can impact speech intelligibility in SiS situations. Among cognitive abilities supporting SiS processing and comprehension, it is suggested that working memory (WM)—one of the executive function (EF) abilities ([17–19], see also [20–22])—is a key component of language comprehension [23–26]. Consistent with this assumption, studies have suggested that a decline in WM capacity in older adults is related to a decline in SiS comprehension [27]. Furthermore, in hearing aid users and hearing impaired people, it has been reported that better WM scores are related to better speech-in-noise performance [28,29]. It was suggested that WM allows matching between linguistic input and phonological representation in semantic long-term memory [30]. In accordance with this assumption, a meta-analysis found that WM measured by reading span is the better cognitive predictor of speech-in-noise recognition performance [31]. However, a recent meta-analysis revealed that, taking into account the different populations tested, for young listeners with audiometrically normal hearing, individual variations in WM would account for around 2% of the variance in speech in noise identification scores [32]. Still, it appears that WM training, consisting of backward digit span training, may improve speech-in-noise perception [33].

Other executive components could be involved in SiS comprehension [34]. In a full three-factor model, EFs were described as a set of three components that all correlated with each other, i.e., updating, switching and inhibition [35]. Updating is closely linked to WM and is a key component of WM abilities [36,37]. It defined as the ability to monitor, code and revise incoming information in WM and to replace irrelevant information with relevant and novel information [38,21,39]. Switching involves shifting back and forth among multiple tasks or mental sets [40]. In a SiS situation, it could be essential to switch between different voice streams [41]. Finally, inhibition refers to the ability to inhibit dominant responses. In a SiS situation, inhibition could be related to selective attention control [42]. Indeed, selective auditory attention is a critical component of speech processing in SiS perception, allowing relevant stream selection and irrelevant stream inhibition [43–45].

In the present study, we explored the effect of the involvement of listeners’ EF ability of semantic processing in SiS situations. Specifically, we evaluated semantic processing in SiS situations via a cross-modal semantic priming paradigm that allow to tackle semantic activation, and we evaluated for each participant various EF processes [35]. To do this, we proposed a classical WM task (the digit span task from the French version of WAIS-IV) that measures working memory capacity and a modified version of the anti-saccade task [42]. This task allowed the simultaneous evaluation of three different components of executive processes: response suppression, inhibitory control (both measuring executive inhibitory abilities) and switching. Thus, if semantic processing is dependent on EF, the priming effect amplitudes should be related to EF performances. We expected to observe a correlation between EF performances (digit span, response suppression, inhibitory control, and switching) and priming effect amplitudes.

Furthermore, in the present study, we also investigated directly the role of auditory attention in the semantic priming effect that, for now, remains unclear. Indeed, several studies have shown that attention increases semantic priming effect [46]. Nevertheless, other studies have stipulated that semantic priming effect observed in unattended condition is due to the ability to switch auditory attention to the unattended condition [47]. Thus, in the cross-modal semantic priming experiment, we manipulated auditory attention by asking participants to listen to only one of the two simultaneously pronounced sentences and, at the same time, to perform a visual lexical decision task on the target stimulus. The prime word was implemented in the attended sentence or in the ignored sentence. Thus, if semantic activation needed auditory attention, we expected to observe a semantic priming effect only when the prime was in the attended sentence and not in the ignored sentence.

## Method

This study have been approved by the Ethics Committee Sud-Est II, and have been conducted according to the principles expressed in the Declaration of Helsinki. No minors were included in the study.

### Semantic priming task

A cross-modal priming paradigm was used in a SiS situation. Participants were instructed to perform a visual lexical decision task while listening to one of two streams of speech. The prime was implemented in one of the two speech streams each consisting of one sentence. One stream was pronounced by a female voice and the other by a male voice. Participants’ attention and the semantic relation between the target and the prime were manipulated. Participants were instructed to pay attention to only one of the two speech streams according to the gender of the voice. The listened-to speech stream contained (attended condition) or did not contain (ignored condition) the prime word, and the prime word was semantically related or semantically unrelated to the visual target word. This allowed us to assess the semantic priming effect (unrelated condition–related condition) and the possible modulation of this semantic priming effect by auditory attention.

#### Participants

Sixty-two psychology students from the University of Grenoble Alpes (46 females, M_age_ = 22 years, SD = 6; 16 males, M_age_ = 21 years, SD = 2) participated in this experiment. All were native French speakers and reported no known language or auditory disorder. All participants gave written informed consent and received course credit for their participation.

#### Stimuli

First, 360 words and 120 pseudo-words (according to the French orthographic rules) were selected. The words included 120 targets, 120 semantically related and 120 unrelated prime words. They were selected from both a preceding study [13] and a verbal association database [48]. Pairs of primes (semantically related and unrelated) were matched in terms of lexical frequency (according to the French database, Lexique 3 [49]) and number of syllables. Table 1 shows the psycholinguistic characteristic of the target and prime words used.

**Table 1:** Psycholinguistic characteristics of word stimuli.

	Lexical Frequency (per million)	Number of Syllables
**Target**	**63.28**	**1.98**
	(125.95)	(0.48)
**Semantically related Prime**	**41.46**	**1.98**
	(100.29)	(0.55)
**Semantically unrelated Prime**	**40.73**	**1.98**
	(92.17)	(0.55)

Table 1 shows the means (in bold) and standard deviations (in parentheses) of psycholinguistic variables, controlled for each type of verbal stimulus.

Second, 840 sentences were constructed and recorded with an OLYMPUS LS100 recorder. To allow participants to perform stream segregation easily, sentences recorded were uttered by one male (mean of F_0_ = 137.27 Hz) and one female voice (mean of F_0_ = 238.21 Hz). Each sentence was composed of 18 syllables and had a comparable duration (for the male speaker, M = 2916 ms, SD = 235; female, M = 3237 ms, SD = 361). Each recorded sentence underwent noise-reduction processing, intensity calibration in dB-A and normalization at 80dB-A. SiS stimuli were built by merging one sentence produce by the male speaker and one produced by the female speaker. Both male and female speakers’ sentences started simultaneously after 200 ms of silence. Sentences were processed with Audacity software (2.1.1. Version, http://www.audacityteam.org/). During the experiment the sound tracks were presented to participants over headphone at 60 dB-A.

Among these 840 sentences, 480 were used for the relevant speech stream (i.e. containing the experimental primes), 240 for the irrelevant speech stream, and 120 for the pseudo-words’ presentation. Half of the 480 sentences used as relevant speech stream contained the semantically related prime, and the other half contained the unrelated one. Half of the time the prime was at the beginning of the sentence (at least 3 syllables after the start of the sentences—e.g., for the target *bequille*, i.e., “crutch”, the semantically related prime was *entorse*, i.e., “sprain”. *Regardez l’****entorse*** *que Nicolas s’est faite en faisant son footing*; “Look at the sprain that Nicolas got from jogging”, and the semantically unrelated prime was *décence*, i.e., “decency”. *Il a la* ***décence*** *de voir sa mère malgré ces rapports avec son père; “*He has the decency to see her mother despite the relationship with his father”). The other half of the sentences contained the prime words further in the sentences (but at least 3 syllables before the end of the sentences—e.g., *Hier*, *Nicolas s’est fait une* ***entorse*** *en allant au parc botanique*; “Yesterday, Nicolas got a sprain while going to the botanical park”, *Malgré ses rapports avec son père*, *il a la* ***décence*** *de voir sa mère*; “Despite his relationship with his father, he has the decency to see her mother”).

Prime position (near the beginning or near the end of the sentences), the gender of the speaker of the speech stream (male and female), attention (attended and ignored) and semantic relatedness (related and unrelated) variables were counterbalanced in sixteen lists of 240 sentences each. Each participant performed only one list. Additionally, 48 supplementary sentences were constructed for the training session.

#### Task and procedure

**Fig 1:**
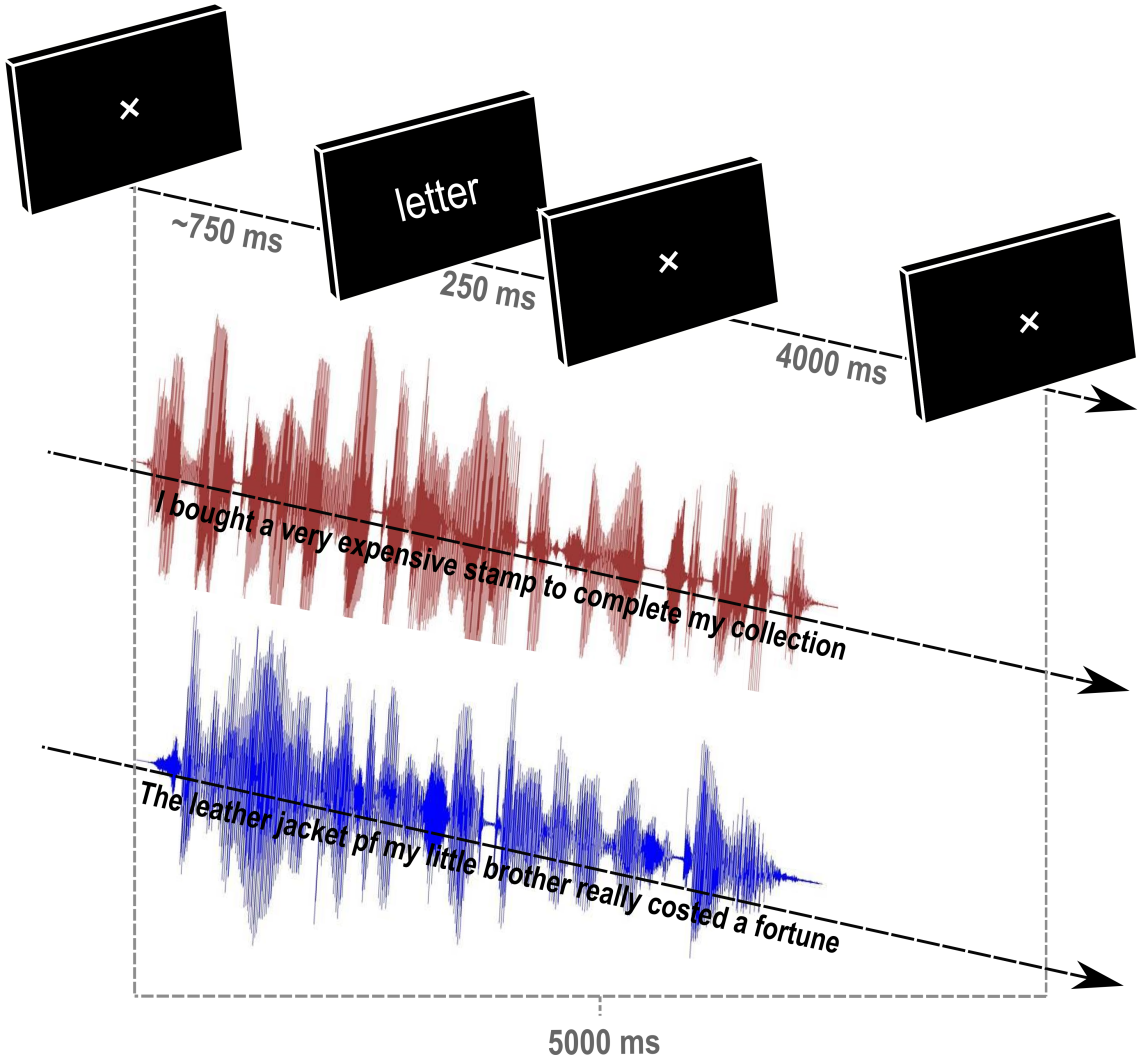
Cross-modal semantic priming paradigm in speech-in-speech situation. An example of one trial, with *letter* as the target written word and *timbre “stamp”* as the auditory prime. In this example, the prime was semantically related to the target (SEMR condition), and the relevant speech stream (A condition) was pronounced by the female (represented by pink speech stream).

Participants were tested individually in a quiet room and sat in front of a black screen. They were instructed to perform a visual lexical decision task, i.e., to decide as quickly and accurately as possible whether the string of characters presented at the center of the screen was a French word or not. Concurrently, they were instructed to pay attention to one of the two auditorily presented sentences of the SiS stimuli (delivered by headphones at 60 dB-A). More specifically, half of the participants were instructed to pay attention to the male voice; the other half, to the female voice. Each participant performed 24 training trials before the 240 experimental trials. One trial was performed as follows (Fig 1): After the beginning of the auditory SiS stimuli, the screen (on which the central fixation point was signaled) remained black until the end of the pronounced prime word. Then, the visual target was displayed for 250 ms, followed by 4000 ms of a black screen. The onset of the visual target items corresponded to the offset of the auditory prime items [10]. Participants indicated their behavioral response manually on keyboard (a right key for “word”; a left key, for “pseudo-word”). Furthermore, to motivate participants to display attentional focus on the correct auditory sentence, we presented all 5 trial check questions (for a total of 48 questions), in which participants indicated whether a given word had been pronounced in the last sentence they had listen. They gave their response using the same key as for the lexical decision.

### Executive function assessment

The Executive Function (EF) assessment was done using a modified version of the anti-saccade task [42]. This task allows the assessment of three different components of two executive processes, namely inhibition and switching. Response-suppression cost and inhibitory control evaluated inhibition, while switching cost evaluated switching. Following Bialystok et al. (2006) [42], we used an adapted version of the anti-saccade task (initially based on eye movements) by collecting the manual response given for the detection of a flashing target appearing randomly on the left or right side of the central fixation region of the screen, in which a schematic face was drawn. The schematic face presented a straight gaze direction (straight-gaze condition) or a changing gaze direction (changed-gaze condition). Participants had to press as quickly and accurately as possible the same side as the target presentation when the eye color was green (PRO condition) and the opposite side when the eye color was red (ANTI condition). The dark flashing target display induced reflexive eye movement to the visual target [50,51].

In the ANTI condition, participants had to suppress the prepotent response (i.e., press the response button on the same side at the target presentation) [52,53] in order to press the response button on the opposite side. The suppression of the prepotent response induced a cost, reflected by an increased reaction time. Thus, a direct comparison between the ANTI and PRO conditions allowed the assessment of the first evaluated executive component, i.e., response-suppression cost (Suppression-cost).

Furthermore, in the changed-gaze condition, the gaze direction of the schematic face was congruent or incongruent with the target presentation side. Gaze direction was an irrelevant information that participants needed to ignore to be able to perform the task correctly. However, humans are sensitive to eye gaze, and it is difficult to resist following gaze direction or a symbolic representation of it [54,55]. In the incongruent condition, participants needed to inhibit the interfering information conveyed by the gaze direction. This induced a cost, reflected by longer reaction times in the incongruent condition than in the congruent condition. Thus, a direct comparison between the incongruent and congruent conditions allowed the assessment of the inhibitory control cost (Inhibition-cost), corresponding to inhibitory control, the second executive component assessed.

Finally, the manipulation of the ANTI and PRO trial presentations in a blocked or mixed manner allowed the evaluation of switching abilities and, more specifically, the assessment of switching cost (Switching-cost). Indeed, in the mixed condition (ANTI and PRO trials randomly presented), participants were required to hold in their minds two sets of instructions and execute the appropriate instructions according to the eye color of the schematic face. This shift between ANTI and PRO trial instructions induced a cost reflected by longer reaction times in the mixed than in the blocked conditions. Thus, a direct comparison between the two conditions allowed the evaluation of switching abilities via the Switching-cost, the third executive component assessed in our study.

#### Participants

Forty-three participants (33 females, M = 21 years, SD = 6 and 10 males, M = 21 years, SD = 2) of the 62 participants who completed the priming task also performed the EF assessment.

#### Stimuli

We constructed 7 different schematically drawn faces that varied in terms of gaze direction and eye color, similar to that used by Bialystok et al. (2006) [42]. Three different types of straight-gazing schematic faces were constructed: one with red eyes, one with green eyes, and one with white eyes (used to indicate the start of the trial). Four different types of changing gaze schematic faces were also constructed: Two faces presented a right gaze direction, one with red and one with green eyes; and two presented a left gaze direction, one with red and one with green eyes.

#### Task and procedure

**Fig 2:**
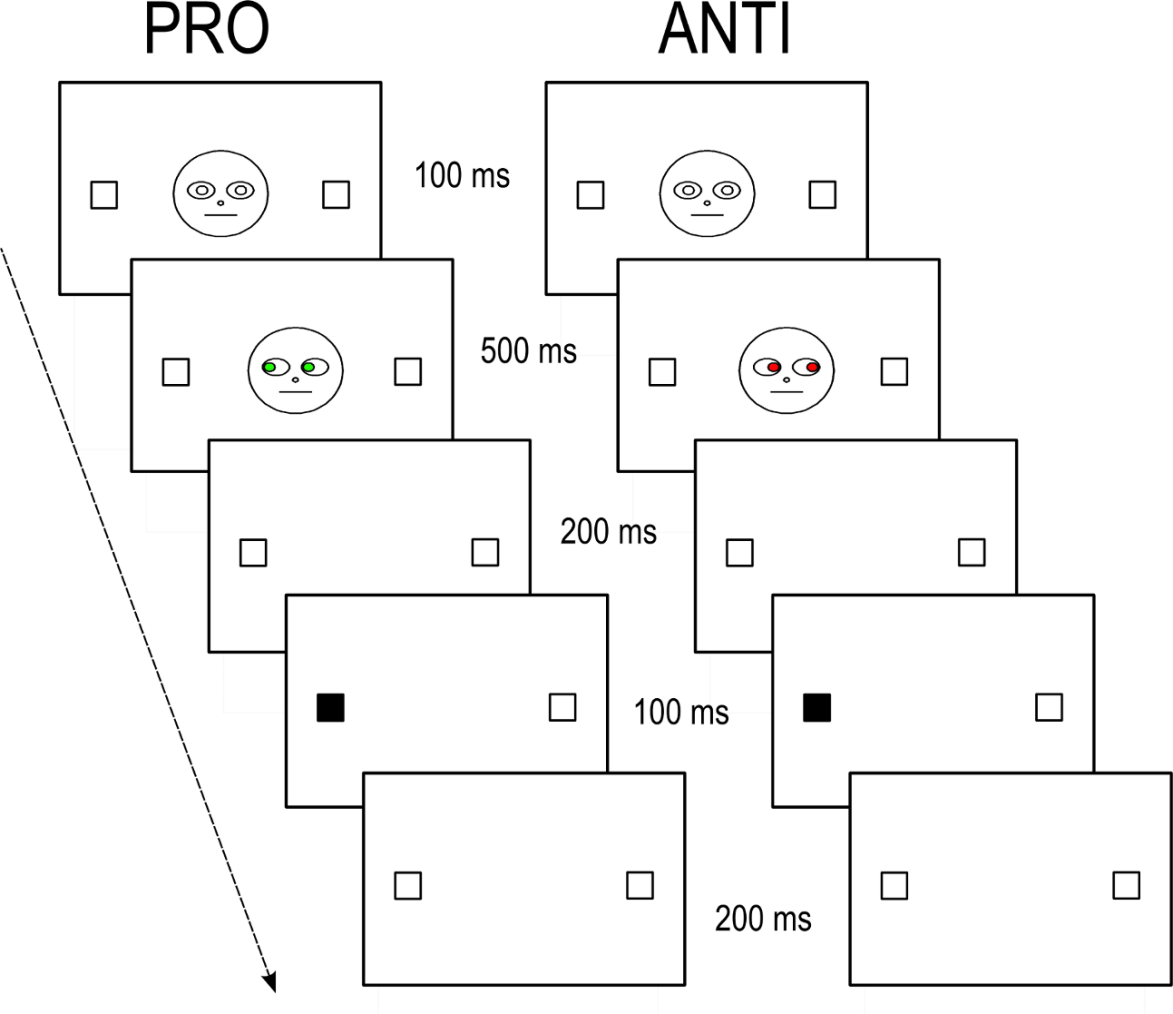
Trials examples of the modified version of the anti-saccade task. A typical trial of the modified anti-saccade task. We present PRO and ANTI trials for the changed-gaze condition. One trial (in the PRO condition) included a gaze direction on the same side as the display side of the target (congruent condition), in which participants were instructed to press the keyboard on the same side of the target. The other trial (in ANTI condition) included a gaze direction on the opposite side of the display side of the target (incongruent condition) in which participants were instructed to press the keyboard on the opposite side of the target.

Each participant was tested individually in a quiet room. They were seated in front of a computer monitor with a white screen. Participants were instructed to detect as quickly and accurately as possible the flashed target by pressing buttons. Each trial (Fig 2) began with the presentation of a schematic face with straight-gazing and white eyes. This schematic face appeared between two empty squares on each side for 1000 ms. Then, the eyes of the schematic face became red or green for 500 ms with a straight, left or right gaze direction, depending on the condition. Then, the schematic face disappeared for 200 ms. Just afterwards, a dark flash was briefly presented (150 ms) in one of the lateralized squares (on the left or right side). After that, the screen remained white with empty lateralized squares for 1350 ms until the next trial. During this time, participants gave their manual responses. If the eye color was green, the task was to respond to the side of the target, and if the eye color was red, the task was to respond to the opposite side of the target. Participants performed 8 blocks of 64 trials, divided into two sessions. One session was composed of only straight-gaze trials; the other, of only changed-gaze trials. Each session was divided in four parts according to ANTI and PRO trials. Two of these parts were blocked: one with only ANTI trials and the other with only PRO trials. The two other parts of each session were presented in a mixed manner: ANTI and PRO trials presented randomly. Specifically, in the changed-gaze session, half of the trials presented the right gaze direction (128 trials); the other half, the left gaze direction (128 trials). Moreover, half of each 128 trials were incongruent (64 trials) with the target position, and the other half were congruent with the target position (64 trials). Conditions were counterbalanced between participants: half of participants began with the straight-gaze condition and the other half with the changed-gaze condition. For each of these conditions, half of the participants began with the mixed presentation, and the other half with the blocked presentation.

### Assessment of working memory capacity

#### Participants

Forty-three participants (33 females, M = 21 years, SD = 6 and 10 males, M = 21 years, SD = 2) of the 62 participants who completed the priming task experiment and the EF task also performed the WM task.

#### Material and stimuli

We used the forward, backward, and ascending digit span task in the working memory task of the French version of WAIS-IV [56].

#### Task and procedure

This test was delivered by the experimenter and included three different sessions according to each of the WM tasks in the French version of the digit span task of the WAIS-IV. For each session, the experimenter repeated the digits of each sequence one by one. The first session was the forward digit span task, in which participants were instructed to repeat each digit sequence in the same order. The second session consisted of the backward digit span task, in which participants were instructed to repeat each digit sequence in reverse order. The third session was the ascending digit span task, in which participants were instructed to repeat each digit sequence in ascending order.

Each session was composed of 8 trials increasing in difficulty (consisting of an increasing number of digits, 2 to 10 digits), and each trial was composed of 2-digit sequences. For the three sessions, the experimenter stopped the presentation when participants performed two consecutive errors in the same trial. For each participant and each session, the WM score consisted of the correct response rate (i.e., the ratio of the number of correctly repeated sequences / the total number of sequence).

## Data analysis

### Semantic priming task

The dataset comported 7440 observations. We excluded two participants; the first one was slow (M = 973 ms, SD = 423) compared with the group mean reaction time (M = 686 ms, SD = 217), and the second one had an error rate that exceeded 15% (M = 16.66% SD = .36), three times greater than the group mean (M = 5.91%, SD = 3.86). Moreover, three items were removed from the dataset because they induced high error rates (more than 40%: *fiasco*, *pagaie*, *tempo*) compared to the other items (M = 4.23%, SD = 4.18). Finally, outliers, i.e., latencies less than 300 ms and greater than 1500 ms, were also removed from data. Overall, a total of 6.64% of the original data was discarded for the data analysis. The mean of RTs for correct responses on the word targets was 621 ms (SD = 75), and there was an ER of 4.06% (SD = 3.13).

Mixed analyses of variance (ANOVA) with participants and items as random variables [57,58] were conducted after verifying the assumption of homogeneity of variance on the RTinv (= -1/RTs*1000) and on the ERs [59–62]. Only responses for target words were considered and included in a 2 x 2 ANOVA with Attention (attended vs ignored) and Semantic relatedness (semantically related vs unrelated) variables as within-participant factors. We expected an interaction between Attention and Semantic relatedness. More precisely, we expected a larger semantic priming effect (i.e., difference between semantically related and unrelated conditions) in the attended condition than in the ignored condition.

### Executive function assessment

For the anti-saccade task, the dataset consisted of 22016 observations. Half of these observations corresponded to the straight-gaze condition; the other half, to the changed-gaze condition. For the straight-gaze condition, 31 observations were absent responses and were consequently removed from the dataset as 4 anticipation responses (RTs lower than 80 ms [42]). Thus, 0.31% of the original straight-gaze condition data were removed from the data analysis. For the changed-gaze condition, 66 absent responses and 17 anticipation responses (corresponding to 0.75% of the initial dataset of the condition) were also removed.

An ANOVA was conducted for each condition (straight-gaze and changed-gaze) in terms of RTs and ERs. The straight-gaze ANOVA included Switching (blocked and mixed conditions) and Response suppression (PRO and ANTI conditions) variables as within-participant factors. The changed-gaze ANOVA included Switching (blocked and mixed conditions), Response suppression (PRO and ANTI conditions) and Inhibitory control (congruent and incongruent conditions) variables as within-participant factors. We expected to observe a significant main effect of Switching (larger RTs in the mixed than in the blocked condition), Response suppression (larger RTs in the ANTI than in the PRO condition) and Inhibitory control (larger RTs in the incongruent than in the congruent condition). Moreover, as previously reported [42], we expected to observe significant interactions among these variables.

### Assessment of working memory capacity

For the WM tasks of the WAIS-IV, we recorded each participant’s correct responses on the digit spans for forward (FDS), backward (BDS) and ascending (ADS) digit span tests. We used the correct response rates in correlation analyses (see below).

### Correlational analyses

If, in the SiS situation, semantic activation depends on the EF abilities of the listener, we should observe correlations between semantic priming effects and EF performances. To evaluate the relationship between semantic activation and EFs in SiS situations, we performed correlation analyses between EF performances and two priming effects, i.e. the one observed in the attended condition and the one observed in the ignored condition. EFs included performances of the anti-saccade task (with the Suppression-cost, the Inhibition-cost, and the Switching-cost) and of the WM task (with the correct response rate on the FDS, the BDS, the ADS tasks, and Global Working Memory Score, i.e., the mean of the three previous scores).

## Results

### Semantic priming task

The results suggest that participants correctly focused their attention in the relevant speech stream, as demonstrated by the performances to the check questions (M = 92.23%; SD = 5.11).

**Fig 3:**
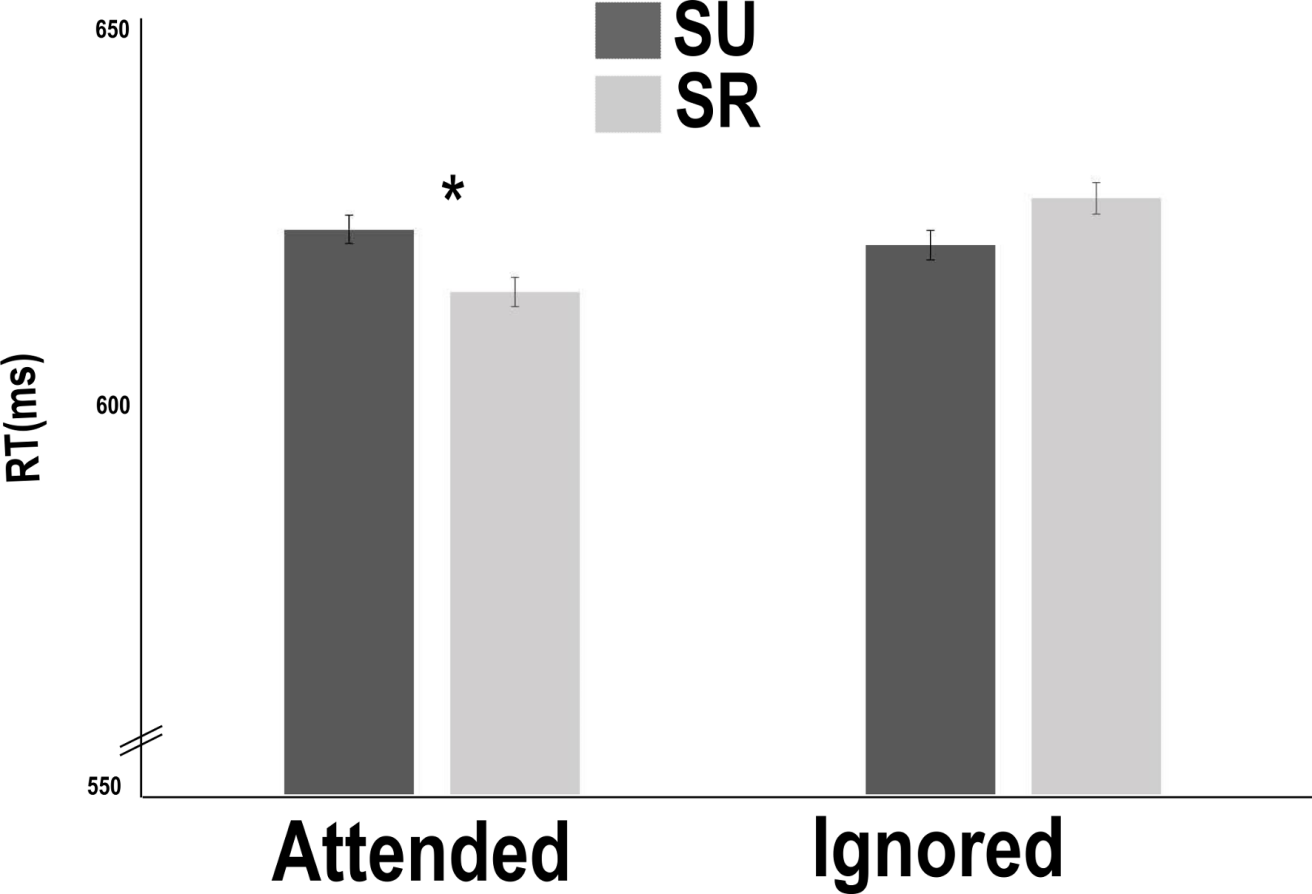
Priming effect results. A significant interaction between attention and semantic priming effect in terms of reaction times. The results showed that in the attended condition, the semantically related (SEMR) items were recognized faster than semantically unrelated (SEMU) items, while this difference was absent for the ignored condition.

The analysis performed on RTs revealed a significant main effect of Attention [*F*(1,6510) = 4.28, *p* < .05], with faster RTs in the attended condition (M = 618 ms, SD = 150) than in the ignored condition (M = 623 ms, SD = 156). The Semantic relatedness main effect was not significant [*F*(1,6501 = .75, *p* = .38]. Indeed, no significant differences in terms of RTs were observed between the semantically related condition (M = 624 ms; SD = 82) and the unrelated one (M = 625 ms; SD = 78). However, the interaction between Attention and Semantic relatedness was significant [*F*(1,6488 = 6.32, *p* < .05] (Fig 3). Planned comparisons showed that a semantic priming effect was significant only in the attended condition [*t*(1,6494) = 2.398, *p* < .05], with an amplitude of 8 ms, (faster RTs in the semantically related condition, M = 614 ms, SD = 152, than in the unrelated one, M = 622 ms, SD = 148).

The analysis performed on the 4.06% ER showed an absence of a main effect of Attention [*F*(1,6939) = 1.02, *p* = .62] and a significant main effect of Semantic relatedness [*F*(1,6939) = 9.78, *p* < .05], with a greater ER when the prime was unrelated (M = 4.80%, SD = 21.35) than when it was semantically related (M = 3.28%, SD = 17.8). The Attention x Semantic relatedness interaction was not significant [*F*(1,6939) = .04, *p* = .84].

### Executive functions

#### Straight-gaze condition

**Fig 4:**
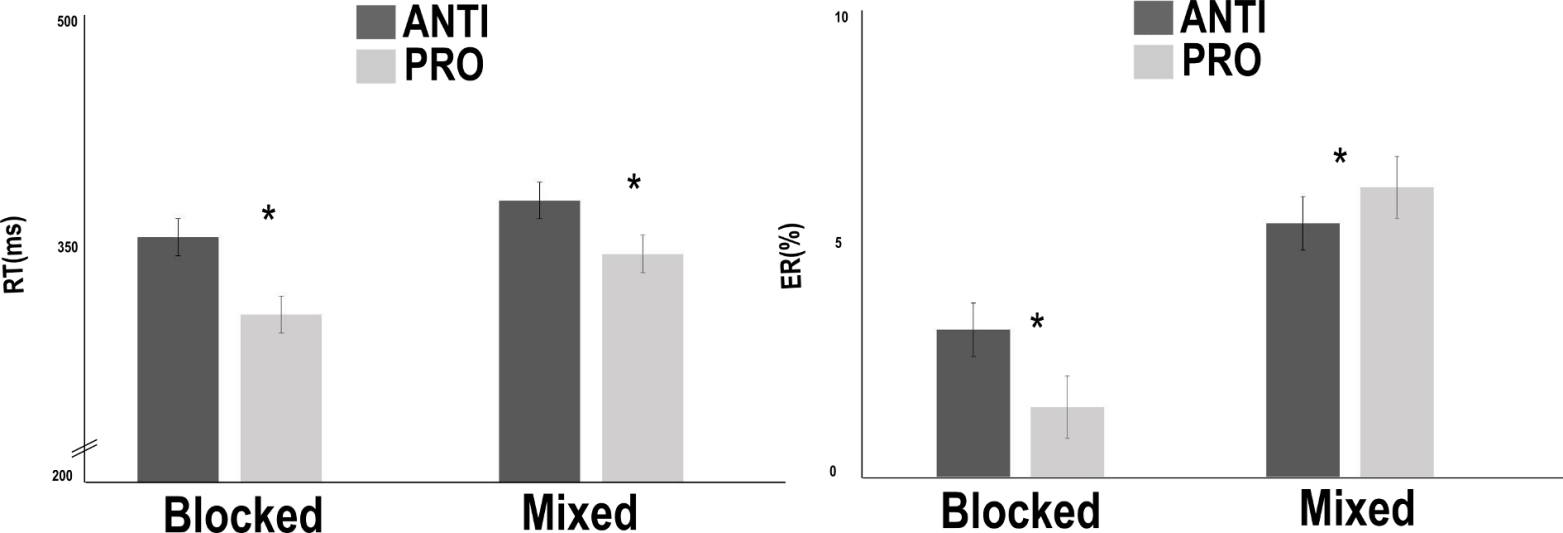
Results of the modified version of the anti-saccade task in the straight-gaze condition. A significant interaction between response suppression and switching in terms of reaction times (RTs). The results showed that in the blocked condition, the differences in the RTs and error rates (ERs) between the ANTI and PRO conditions were greater than in mixed condition.

In terms of RTs, the results showed a switching cost of 32 ms. Indeed, there were significantly [*F*(1,42) = 30.117, *p* < .05] faster responses in the blocked condition (M = 336 ms, SD = 56) than in the mixed condition (M = 368 ms, SD = 78). The results also showed a response-suppression cost of 43 ms, with significantly [*F*(1,42) = 160.08, *p* < .05] faster responses in the PRO condition (M = 331 ms, SD = 67) than in the ANTI condition (M = 373 ms, SD = 67). The Switching x Response-suppression interaction was also significant [*F*(1,42) = 9.563, *p* < .05], and the response-suppression cost was greater in the blocked condition (M = 52 ms, SD = 30) than in the mixed condition (M = 35 ms, SD = 27) (Fig 4).

The analysis of the 4.09% ER revealed a switching cost of 3.51%. Indeed, there were significantly greater [*F*(1,42) = 53.65, *p* < .05] ERs in the mixed condition (M = 5.84%, SD = 3.6) than in the blocked condition (M = 2.33%, SD = 1.78). An absence of a response-suppression cost was observed [*F*(1,42) = 1.17, *p* = .28]. The Switching x Response-suppression interaction (see Fig 4) was significant [*F*(1,42) = 12.09, *p* < .05], and show, as for RTs, a greater response-suppression cost in the blocked condition (M = 1.67%, SD = 3.06) than in the mixed condition (M = 0.77%, SD = 4.02).

#### Changed-gaze condition

**Fig 5:**
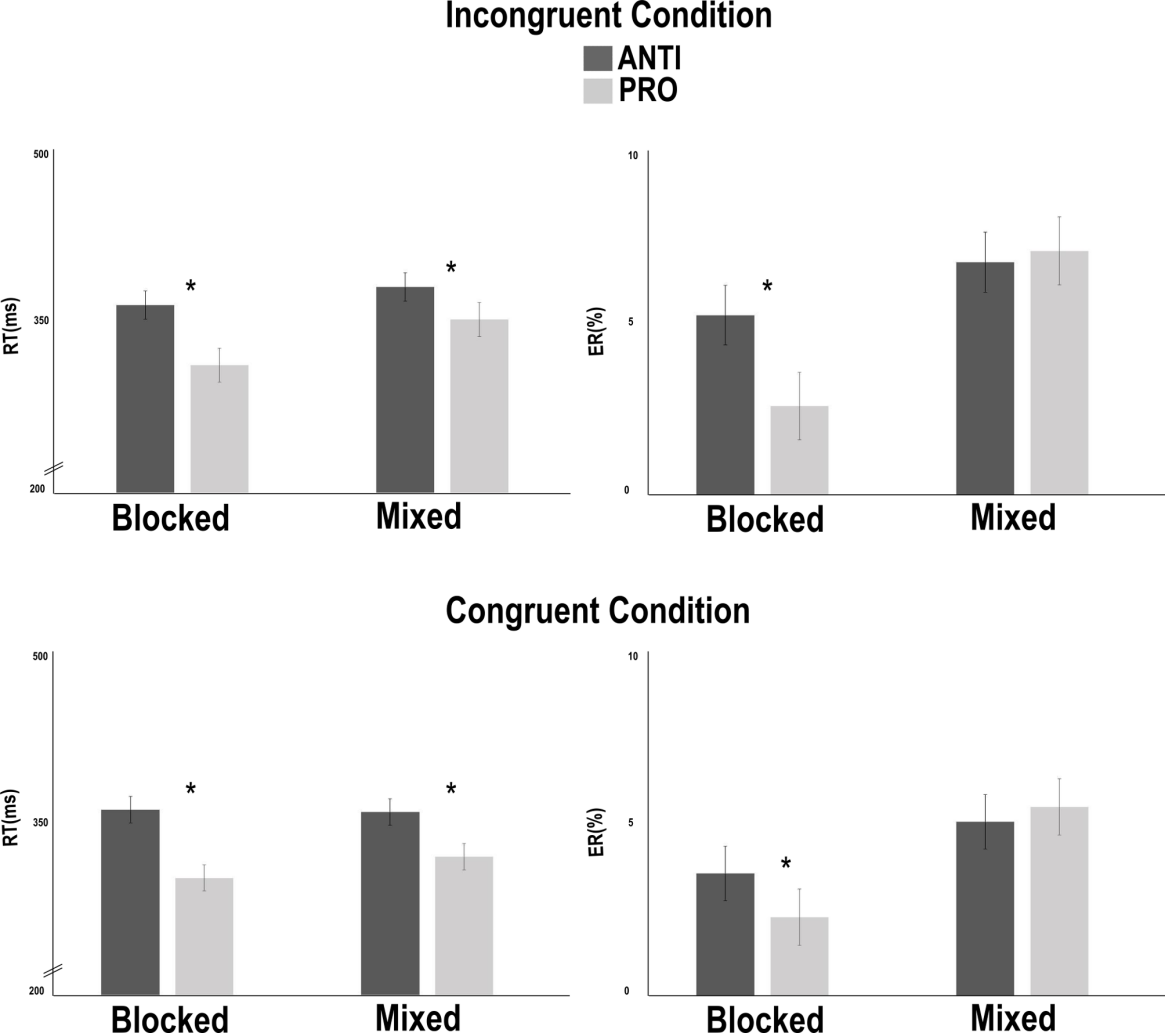
Results of the modified version of the anti-saccade task in the changed-gaze condition. Switching x response-suppression interaction in incongruent and congruent trials. The results showed that in the blocked condition, the differences in RTs and error rates (ERs) between ANTI and PRO conditions were greater than in mixed condition.

The RT analyses revealed a Switching cost of 18 ms, with significantly [*F* (1,42) = 14.1, p < .05] faster responses in the blocked condition (M = 338 ms, SD = 64) than in the mixed condition (M = 356 ms, SD = 82). The results also showed a Response-suppression cost of 46 ms, with significantly [*F*(1,42 = 163.91, *p* < .05] faster responses in the PRO condition (M = 324 ms, SD = 74) than in the ANTI condition (M = 370 ms, SD = 71). Furthermore, we observed an Inhibitory-control cost of 16 ms, with significantly [*F*(1,42) = 28.10, *p* < .05] faster responses in the congruent condition (M = 339 ms, SD = 70) than in the incongruent condition (M = 355 ms, SD = 75). The results also showed a significant interaction of Switching x Response suppression (*F*(1,42) = 18.313, *p* < .05), with a greater response-suppression cost in the blocked condition (M = 58 ms, SD = 35) than in the mixed condition (M = 34 ms, SD = 24) (Fig 5). The Switching x Inhibitory control interaction was also significant [*F*(1,42) = 16.371, *p* < .05), showing a greater inhibitory-control cost in the mixed condition (M = 26 ms, SD = 30) than in the blocked condition (M = 5 ms, SD = 20).

Analyses of the 4.87% ER revealed a Switching cost of 2.76%, with significantly [*F*(1,42) = 24.02, *p* < .05] greater ERs in the mixed condition (M = 6.25%, SD = 4.30) than in the blocked condition (M = 3.49%, SD = 2.87). The results also showed a lack of effect [*F*(1,42) = 2.36, *p* = .13] of the Response-suppression cost. Furthermore, we observed an Inhibitory-control cost of 1.34%, with significantly greater [*F*(1,42) = 10.69, *p* < .05] ERs in the incongruent condition (M = 5.55%, SD = 3.45) than in the congruent condition (M = 4.20%, SD = 3.41). Finally, only the Switching x Response-suppression interaction was significant [*F*(1,42) = 7.32, *p* < .05], showing a greater response-suppression cost in the blocked condition (M = 2.00%, SD = 3.17) than in the mixed condition (M = -0.39%, SD = 5.51).

### Working memory task

The Table 2 shows the WM mean scores in terms of the correct response rate and maximal digit span obtained in the three subtests (FDS, BDS, and ADS) and the global working memory score—GWMS (i.e., means of FDS, BDS and ADS scores).

**Table 2:** Working memory scores.

	FDS	BDS	ADS	GWMS
**Mean of rate of correct response**	**.593**	**.534**	**.61**	**.58**
**(SD)**	(.12)	(.12)	(.11)	(.09)
**Mean of maximal digit span**	**6.25**	**4.93**	**6.65**	**5.94**
**(SD)**	(1.07)	(1.2)	(1.09)	(.87)

Forward, backward, and ascending digit span tests for the mean rate of correct responses and the mean of the digit span conditions. Standard deviations (SD) are presented in parentheses.

### Correlational analyses

**Fig 6:**
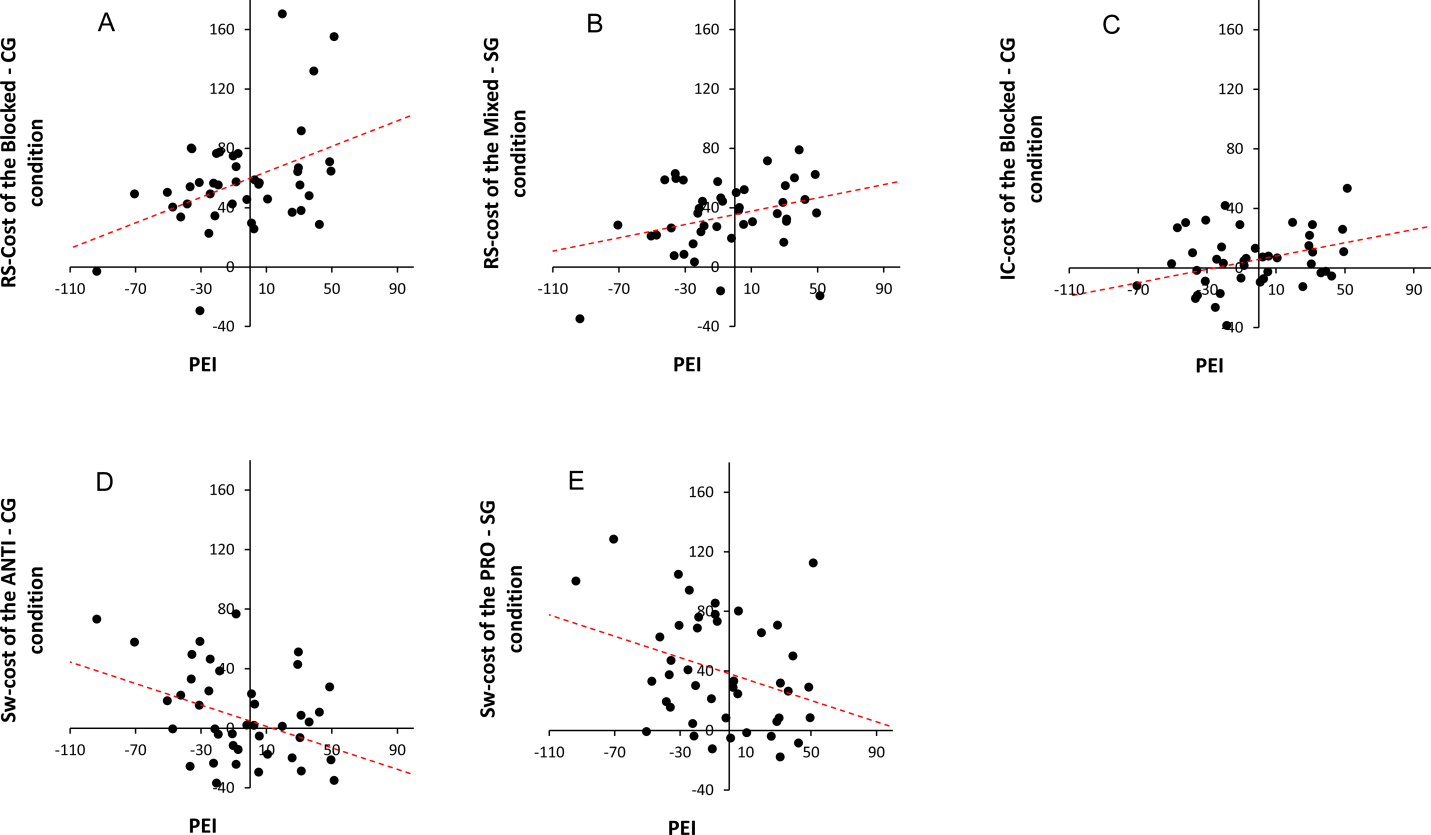
Significant correlations. Correlation results between priming effects in the ignored condition (in abscissa) and performances (in ordinate) for: **(A)** the changed-gaze blocked-Suppression-cost; (**B)** the straight-gaze mixed-Suppression-cost; (**C)** the changed-gaze blocked-Inhibition-cost; (**D)** the changed-gaze ANTI-Switching-Cost; **(E)** the straight-gaze PRO-Switching-Cost.

To investigate the EF and semantic priming relationship in the SiS situation, we performed correlation analyses of EF performances and the semantic priming effects. These correlations were evaluated according to auditory attention (attended or ignored). Two semantic priming effects were used: the one obtained in the attended condition and the one obtained in the ignored condition. Furthermore, as revealed in the EF results, several performances interacted with each other (i.e., response suppression x switching in both straight-gaze and changed-gaze conditions and switching x inhibition control in the changed-gaze condition). Therefore, we performed a correlation analysis between the semantic priming effects and each of the cross conditions of these interactions. As shown in Table 3, this included, for the priming effect observed in the attended condition and for the priming effect observed in the ignored condition, height conditions of EF: the response-suppression cost in the blocked and mixed conditions, the switching cost in the ANTI and PRO conditions, and the inhibitory-control cost in the blocked and mixed conditions. Scatter plots for significant correlations are presented in Fig 6.

**Table 3:** Correlation analyses results.

		Suppression-cost		Switching-cost		Inhibition-cost	
		Blocked	Mixed	ANTI	PRO	Blocked	Mixed
**STRAIGHT-GAZE**	**Attended Priming**	.013	.189	-.025	-.025	NA	NA
		(.93)	(.22)	(.87)	(.87)		
	**Ignored Priming**	-.184	.217	-.016	**-.319**	NA	NA
		(.24)	(.16)	(.92)	**(.037)**		
**CHANGED-GAZE**	**Attended Priming**	.233	.192	-.025	.066	-.089	-.071
		(.13)	(.22)	(.87)	(.67)	(.57)	(.65)
	**Ignored Priming**	**.420**	**.318**	**-.372**	-.129	**.373**	-.246
		**(.005)**	**(.037)**	**(.014)**	(.40)	**(.014)**	(.11)

Table 3 Shows the correlation results observed between the priming effect (according to attention: priming effect observed in the attended condition and the priming effect observed in the ignored condition) and EF performance, specifically in *i)* the response-suppression cost for the blocked and mixed trials, *ii)* the switching cost in the ANTI and PRO trials, and *iii)* the inhibitory-control cost for the blocked and mixed trials (only for the changed-gaze condition). NA corresponds to not assess.

As shown in Table 3, the correlation analysis for the straight-gaze condition showed a significant correlation only between semantic priming effects in the ignored condition and the switching cost in the PRO condition of the response suppression variable (*r* = -.319, *R*^*2*^ = .102, *p* < .05).

For the changed-gaze condition, the correlation analysis showed that the semantic priming effects in the ignored condition correlated with several EF performances. Specifically, we observed three significant correlations: with the response-suppression cost (in the blocked and mixed conditions: *r* = .42, *R*^*2*^ = .178, *p* < .05 and *r* = .318, *R*^*2*^ = .101, *p* < .05, respectively), with the inhibitory control cost only in the blocked condition (*r* = .373; *R*^*2*^ = .139, *p* < .05) and with the switching cost observed in the ANTI condition of the response-suppression variable (*r* = -.372, *R*^*2*^ = .138, *p* < .05). No other correlations were significant neither in the ignored condition nor in the attended one.

Finally, we observed an absence of a significant correlation between WM scores and the semantic priming effects.

## Discussion

The aim of this study was to investigate the role of EFs in semantic processing in speech-in-speech situations. To do so, we constructed a cross-modal semantic priming paradigm in which we asked participants to pay attention to only one of two simultaneously pronounced sentences and, at the same time, to perform a lexical decision task on visual target item. The auditory prime (implemented in one of the two sentences) and the visual target words were semantically related or unrelated. Thus, semantic activation was evaluated through a cross-modal semantic priming effect. Participants’ attention was manipulated by the gender of the speaking voice. Participants were instructed to pay attention to one sentence pronounced by a male or female voice, that contained or not the prime, and to ignore the other sentence. In addition, we measured each participant’s executive function capacities (by measuring response-suppression cost, switching cost, inhibitory-control cost, and WM capacity) using a modified version of the anti-saccade task proposed by Bialystok et al. (2006) [42] and the digit span task of a French version of WAIS IV. To establish the link between EFs and semantic priming effects, we performed correlation analyses between the semantic priming effects and EF performances.

The results showed, first, an effect of attention on the semantic priming effect. Indeed, as expected, we observed a semantic priming effect when the prime word was in the attended speech stream, whereas the semantic priming effect was absent when the prime word was in the ignored speech stream. This result suggests greater difficulty in semantic access and processing in ignored speech stream than in attended speech stream. This result was consistent with several previous results observed in semantic priming paradigms showing that the priming effect required attention to be observed [8,46,47, 63]. Moreover it suggests that activation of semantic representation is not irrepressible and that semantic processing in a SiS situation seems to depend on the limited capacity of auditory attention [13].

Correlation analyses revealed that there was no correlation between EF performances and the semantic priming effect in the attended condition, suggesting that when the listener is focused on the speech signal, semantic processing does not seem to be linked to inter-individual EF differences, at least in the SiS situation composed of two speech streams (at 0 SNR), in which participants were able to correctly focus their attention on the relevant speech stream with relative ease. Interestingly, our results showed that in the ignored condition, the three EF performances (i.e., response-suppression cost, switching cost and inhibitory-control cost) obtained in the changing gaze condition correlated with the semantic priming effect. It appears indeed that inhibitory capacities are correlated with the semantic priming effect observed in the ignored condition: the greater the response-suppression cost, the greater the semantic priming effect and the greater the inhibition-cost, the greater the semantic priming effect. In other words, the lower inhibition capacities were, the more the ignored speech stream was semantically processed. Response suppression and inhibitory control are related to the inhibitory capacities that correspond to the ability to control the prepotent response and to prevent acting on irrelevant information. Our results showed that when inhibition capacities are suboptimal (corresponding to a high suppression-cost and a high inhibition-cost), participants activated more semantic information of the irrelevant speech stream. These results clearly show the implication of the inhibitory component of EFs in SiS processing and are in line with previous findings suggesting that an active inhibitory mechanism is required on irrelevant/interfering information in selective auditory attention processing [45,64–66].

Furthermore, for the straight-gaze and changed-gaze conditions, the semantic priming effect in the ignored condition correlated negatively with switching abilities, evaluated with the switching-cost in the PRO condition for straight-gaze and in the ANTI condition for the changed-gaze condition. In both cases, these correlations were negative, showing that when switching costs were important, i.e., reflecting poor switching capacities, smaller semantic priming effects were observed. This suggested that when participants paid attention to a speech stream, the size of a semantic priming effect observed on the unattended speech stream depended on the switching abilities of the listeners. More important semantic priming effects were observed for participants with the most efficient switching abilities. This result is consistent with the idea that the semantic priming effect obtained in the ignored condition was underlined by the shift in auditory attention from the attended channel to the ignored channel [47,63]. These results are in line with the idea that switching abilities are strongly involved and very useful during the processing of complex auditory scenes and in particular in speech-in-noise and speech-in-speech situations [34].

The question that remains is why we didn’t observe a correlation between the semantic priming effect in the ignored condition and the three EFs in all conditions. We did not observe, for example, a correlation between response suppression and priming effect in the straight-gaze condition, in contrast to the changed-gaze condition, and while we observed a significant correlation between priming effect and inhibition-cost in the blocked condition we didn’t in the mixed condition. One possibility could be find in the interactions between effects. There were interactions between response suppression and switching variables for both the straight-gaze and changed-gaze conditions and between inhibitory control and switching in the changed-gaze condition. These interactions could explain the fact that we did not observe a significant correlation for all the different measurements of a given executive component. A way to test this assumption could be to use tests measuring only one executive component at a time, for example, the Stroop task for inhibition and TMT for switching capacities.

Another surprising result is the absence of a correlation between the semantic priming effect and WM capacity. Indeed, this is not consistent with most previous findings suggesting that WM abilities are involved and partially explain SiS perception performance [27–29]. The lack of a correlation between WM capacity and semantic priming in our study might be explained by the selection of the digit span task of the WAIS to evaluate WM abilities. Indeed, the digit span may be not the most appropriate task (despite the fact that training in digit span leads to an increase in speech in noise capacities [33]). The first reason is that it was the experimenter who delivered the digit list, which could induce several between-participant variabilities in the stimuli comprehension [67,68]. Moreover as the experimenter stopped the test when the participant missed two-digit lists of the same length regardless of whether the participant achieved on the following lists, it could be that the digit span of WAIS conflates performance with limits in maximal digit span [69] and may not establish a sufficiently precise discrimination of WM capacities between participants. However as suggested by a very interesting recent meta-analysis [32], that surveyed published and unpublished studies using the Reading-span test, it could also be that for young normal hearing participants individual variations in WM are just not sufficient to show up in a correlational analysis. Anyway, as suggest by the Füllgrabe and Rosen [32], these results should not to be interpreted as evidence against the involvement of WM in speech and language processing in young normal hearing adults, as it could be that WM involvement may need, to be visible, more linguistically complex tasks, such as the comprehension of conversations ([70] but see [71] for contrary results for the comprehension of narratives).

To conclude, this study explored the implication of executive functions in speech-in-speech situation processing by measuring *i)* the semantic activation of both attended and ignored simultaneously pronounced sentences, *ii)* the executive functions, and the working memory digit span. Our study revealed an effective relationship between executive function performances and semantic processing in even simple speech-in-speech situations (2 speakers at a 0 SNR). Our results showed that the semantic processing of unattended speech is linked with switching and inhibitory control capacities. This suggests that in speech-in-speech perception, executive functions are widely involved in mitigating unattended speech stream and that switching and inhibitory control abilities could be a prerequisite for speech-in-speech processing.

## Supporting information

S1 File Dataset. Data used for the analyses presented in this paper.
(XLSX)
